# Association between immunoglobulin A and depression in Chinese older adults: findings from a cross-sectional study

**DOI:** 10.1186/s12979-022-00283-y

**Published:** 2022-05-23

**Authors:** Zhigao Sun, Jieqiong Lin, Yujie Zhang, Yao Yao, Zhenjun Huang, Yali Zhao, Pei Zhang, Shihui Fu

**Affiliations:** 1 Traditional Chinese Medicine Department, Hainan Hospital of Chinese People’s Liberation Army General Hospital, Sanya, China; 2 Department of Pathology, Fujian Medical University Cancer Hospital, Fujian Cancer Hospital, Fuzhou, China; 3 Department of Epidemiology, School of Public Health, Southern Medical University, Guangzhou, China; 4 Center for the Study of Aging and Human Development and Geriatrics Division, Medical School of Duke University, Durham, NC USA; 5 Center for Healthy Aging and Development Studies, National School of Development, Peking University, Beijing, China; 6 Central Laboratory, Hainan Hospital of Chinese People’s Liberation Army General Hospital, Sanya, China; 7 School of Life Science, Beijing Institute of Technology, Beijing, China; 8 Department of Cardiology, Hainan Hospital of Chinese People’s Liberation Army General Hospital, Sanya, China; 9 Department of Geriatric Cardiology, Chinese People’s Liberation Army General Hospital, Beijing, China

**Keywords:** China, Depression, Older adults, GDS-15, Immunoglobulin A

## Abstract

**Background:**

Depression is considered to be an immune-related disease; however, previous studies have focused on inflammatory factors, and there is no conclusive conclusion on the relationships between immunoglobulins and depression. Therefore, the objective of this cross-sectional study was to evaluate the associations between immunoglobulins and depressive symptoms in Chinese older adults.

**Results:**

The China Hainan Centenarian Cohort Study (CHCCS) provides a significant population-based sample of older adults in Hainan, China. A total of 1547 older adults were included in this study. A baseline survey was conducted using a structured questionnaire. Blood samples were obtained following standard procedures. The Geriatric Depression Scale (GDS-15) was used to evaluate depressive symptoms of the participants. This sample of older adults had a median age of 94.75 (range: 80–116) years, and the proportion of women was 72.07%. The prevalence of older adults with depressive symptoms was 20.36% (315 older adults). After adjusting for all covariates, we found that immunoglobulin A levels were positively associated with depression. The adjusted reliability of the association between immunoglobulin A and depression was 0.106 (beta) and 1.083 (odds ratio) (*P* < 0.05 for both).

**Conclusions:**

The present study provides epidemiological evidence that depression has significant associations with immunoglobulin A levels in older adults. Further research should be conducted on the effects of regulating immunoglobulin A to improve depressive symptoms.

## Background

Depression is the main cause of suicide, with alterations in mood and emotion as the main clinical features [1, 2]. According to World Health Organization reports, major depression will rank as the leading cause of disease burden worldwide by 2030 [3]. Recent studies have found that the vicious cycle of "mental-nerve-immunity" is an important pathway regarding the occurrence and aggravation of depressive symptoms, which is mainly manifested by abnormal levels of immune factors [4]. Among these factors, immunoglobulin A exerts a variety of anti-inflammatory effects by interfering with the complement innate immune system and inhibiting the release of proinflammatory cytokines [5, 6]. Abnormal levels of immunoglobulin A could disrupt the balance between proinflammatory and anti-inflammatory reactions in the body, which is generally believed to be closely related to the occurrence of depression; however, there have been some differences in specific conclusions [7, 8]. Takagi and Ohira [9] found that patients with depression have higher levels of immunoglobulin A in the saliva. However, Kawano and Emori [10] reported that breast milk solute immunoglobulin A levels were negatively correlated with emotional abnormalities such as depression and anxiety in patients with postpartum depression.

Previous studies have found that old age is an independent risk factor for depression [11]. Researchers [12, 13] have realized that immunoglobulin A levels were closely associated with age in older adults. Immunoglobulin A levels were found to increase with age in a study involving 8768 participants with a median age of 62 years [14]. However, there is little evidence regarding whether immunoglobulin levels are related to depression in older adults. In previous studies, immunoglobulin A levels were lower in Caucasians and showed no significant differences across other ethnicities [12–14]. In addition, data on the associations between immunoglobulins and depression in Asian populations are scarce, especially for Chinese older adults. Therefore, using the database from the China Hainan Centenarian Cohort Study (CHCCS), this cross-sectional study aimed to identify the associations between immunoglobulins and depression in Chinese older adults to provide a reference for the prevention and treatment of depression.

## Results

**Fig. 1:**
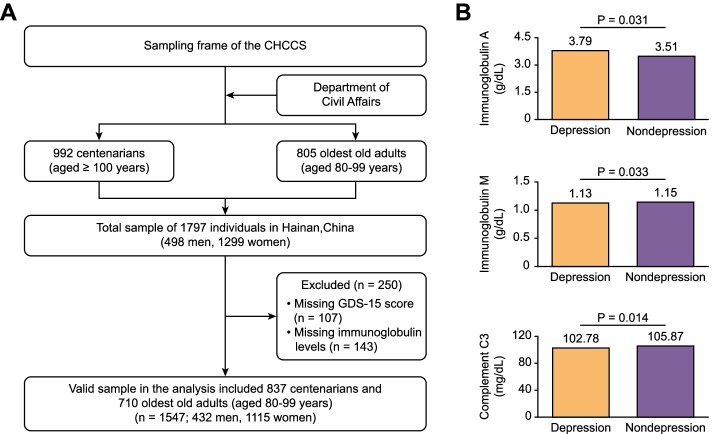
**a** The flowchart of participants in this study; **b** Immunoglobulin A, immunoglobulin M and complement C3 levels in older adults with and without depression

This study included 1115 women (72.07%) and 432 men (27.93%). These older adults had a median age of 94.75 (range: 80–116) years. The prevalence of depression in the total sample was 20.36% (315 older adults). The mean ± standard deviation levels of immunoglobulin A were 3.79 ± 2.29 g/dL for the depression group and 3.51 ± 1.57 g/dL for the nondepression group (*P* < 0.001; Fig. 1b). As Table 1 shows, the group of older adults with depression were older, had a higher percentage of women, had higher levels of immunoglobulin A, and had lower levels of RBC, hemoglobin, immunoglobulin M and complement C3 than the group without depression (*P* < 0.05 for all).

**Table 1:** Characteristics of all participants with and without depression

Variables	Total Sample (*n* = 1547)	Depression (*n* = 315)	Nondepression (*n* = 1232)	*P*
Centenarians (%)	837 (54.10%)	247 (78.41%)	590 (47.89%)	< 0.001
Age (year)^a^	94.75 ± 9.46	99.18 ± 7.85	93.62 ± 9.51	< 0.001
Women (%)	1115 (72.07%)	270 (85.71%)	845 (68.59%)	< 0.001
Ethnic Han (%)	1388 (89.72%)	278 (88.25%)	1110 (90.09%)	0.336
SBP (mmHg)^a^	150.91 ± 24.59	152.06 ± 24.94	150.62 ± 24.51	0.269
DBP (mmHg)^a^	77.97 ± 13.40	77.45 ± 13.68	78.10 ± 13.34	0.476
RBC (10^12^/L)^a^	4.20 ± 0.66	4.00 ± 0.63	4.25 ± 0.66	< 0.001
Hemoglobin (g/L)^a^	119.03 ± 18.28	112.73 ± 18.04	120.65 ± 18.00	< 0.001
MCHC (g/L)^a^	313.46 ± 13.13	312.44 ± 13.16	313.72 ± 13.12	0.140
WBC (10^9^/L)^a^	6.21 ± 1.77	6.14 ± 1.69	6.23 ± 1.79	0.334
Neutrophil^a^	0.56 ± 0.17	0.57 ± 0.11	0.56 ± 0.18	0.229
CRP (mg/L)^a^	0.57 ± 2.06	0.71 ± 1.71	0.53 ± 2.14	0.070
Immunoglobulin A (g/dL)^a^	3.57 ± 1.74	3.79 ± 2.29	3.51 ± 1.57	0.031
Immunoglobulin G (g/dL)^a^	15.91 ± 3.82	16.11 ± 4.11	15.86 ± 3.75	0.579
Immunoglobulin M (g/dL)^a^	1.14 ± 0.65	1.13 ± 0.82	1.15 ± 0.60	0.033
Immunoglobulin E (g/dL)^a^	9.71 ± 30.57	8.91 ± 17.94	9.92 ± 33.03	0.121
Complement C3 (mg/dL)^a^	105.24 ± 24.13	102.78 ± 21.62	105.87 ± 24.70	0.014
Complement C4 (mg/dL)^a^	25.91 ± 9.05	25.36 ± 8.47	26.05 ± 9.20	0.268

*Notes*: ^a^ mean ± standard deviation

*Abbreviations*: *SBP* Systolic blood pressure, *DBP* Diastolic blood pressure, *RBC* Red blood cell, *MCHC* Mean corpuscular hemoglobin concentration, *WBC* White blood cell, *CRP* C-reactive protein

By multivariate linear regression analyses, we found that immunoglobulin A levels were positively associated with Geriatric Depression Scale (GDS-15) (*P* < 0.05 for all, Table 2). The beta value for immunoglobulin A after adjusting for all covariates was 0.106 (95% CI: 0.016–0.195, *P* < 0.05). Table 3 shows the results from multivariate logistic regression analyses. The participants who had higher immunoglobulin A levels tended to have higher odds of depression (P < 0.05 for all). The adjusted odds ratio between immunoglobulin A and depressive symptoms after adjusting for all covariates was 1.083 (95% CI: 1.001–1.172, *P* < 0.05).

**Table 2:** Multiple linear regression analyses between immunoglobulins and GDS-15

Variables	Beta	95% CI	P
Immunoglobulin A			
Model 1	0.118	0.032–0.203	0.007
Model 2	0.102	0.019–0.185	0.016
Model 3	0.106	0.016–0.195	0.020
Immunoglobulin G			
Model 1	0.030	-0.010–0.069	0.140
Model 2	0.000	-0.038–0.038	0.988
Model 3	-0.011	-0.052–0.029	0.585
Immunoglobulin M			
Model 1	-0.271	-0.500- -0.042	0.020
Model 2	-0.248	-0.469- -0.027	0.028
Model 3	-0.315	-0.569- -0.061	0.015
Immunoglobulin E			
Model 1	0.000	-0.005–0.005	0.924
Model 2	-0.001	-0.006–0.003	0.580
Model 3	-0.003	-0.008–0.002	0.253
Complement C3			
Model 1	-0.008	-0.015- -0.002	0.009
Model 2	-0.002	-0.008–0.004	0.567
Model 3	-8.82E-5	-0.008–0.007	0.982
Complement C4			
Model 1	-0.012	-0.028–0.005	0.159
Model 2	0.004	-0.012–0.021	0.595
Model 3	0.004	-0.015–0.023	0.669

*Notes*: Model 1: no adjustment; Model 2: adjusted for age, sex, and ethnicity; Model 3: age, sex, ethnicity, centenarians, SBP, DBP, RBC, hemoglobin, MCHC, WBC, neutrophil, CRP, immunoglobulin A, immunoglobulin G, immunoglobulin M, immunoglobulin E, complement C3, and complement C4

*Abbreviations*: *CI* Confidence interval, *SBP* Systolic blood pressure, *DBP* Diastolic blood pressure, *RBC* Red blood cell, *MCHC* Mean corpuscular hemoglobin concentration, *WBC* White blood cell, *CRP* C-reactive protein

**Table 3:** Multiple logistic regression analyses between immunoglobulins and depression

Variables	OR	95% CI	P
Immunoglobulin A			
Model 1	1.083	1.013–1.157	0.019
Model 2	1.078	1.004–1.157	0.037
Model 3	1.083	1.001–1.172	0.047
Immunoglobulin G			
Model 1	1.017	0.985–1.049	0.307
Model 2	0.995	0.963–1.029	0.782
Model 3	0.988	0.953–1.024	0.513
Immunoglobulin M			
Model 1	0.949	0.780–1.156	0.604
Model 2	0.970	0.793–1.185	0.764
Model 3	0.928	0.729–1.181	0.544
Immunoglobulin E			
Model 1	0.999	0.994–1.003	0.605
Model 2	0.997	0.992–1.003	0.336
Model 3	0.995	0.988–1.002	0.158
Complement C3			
Model 1	0.995	0.989–1.000	0.043
Model 2	1.000	0.994–1.005	0.886
Model 3	1.003	0.995–1.010	0.491
Complement C4			
Model 1	0.991	0.977–1.005	0.223
Model 2	1.003	0.989–1.018	0.653
Model 3	1.001	0.983–1.019	0.929

*Notes*: Model 1: no adjustment; Model 2: adjusted for age, sex, and ethnicity; Model 3: age, sex, ethnicity, centenarians, SBP, DBP, RBC, hemoglobin, MCHC, WBC, neutrophil, CRP, immunoglobulin A, immunoglobulin G, immunoglobulin M, immunoglobulin E, complement C3, and complement C4

*Abbreviations*: *OR* Odds ratio, *CI* Confidence interval, *SBP* Systolic blood pressure, *DBP* Diastolic blood pressure, *RBC* Red blood cell, *MCHC* Mean corpuscular hemoglobin concentration, *WBC* White blood cell, *CRP* C-reactive protein

## Discussion

In our study, older women reported depressive symptoms more frequently than older men, and there was a significant correlation between immunoglobulin A levels and depression. After adjusting for the covariates, this association remained significant. Based on this study, we confirmed that immunoglobulin A is a good biomarker that could be used to effectively identify those with depression and, the mechanism involved in regulating immunoglobulin A levels might be responsible for the development of depression.

It is well known that immunoglobulins are closely related to the occurrence of depressive symptoms in specific populations [15, 16]. Denitsa revealed that major depression was accompanied by higher serum immunoglobulin M/A responses, and compared with controls, depressive patients showed higher immunoglobulin A responses to *Citrobacter koseri*, which might be related to the microbe-gut-brain axis [4]. Francois found that immunoglobulin M levels were positively correlated with girls’ anxiety scores among 1199 adolescents (636 girls) in the Netherlands [17]. Another study involving mothers with major depression and their children revealed that mothers had higher levels of solute immunoglobulin A in their saliva and showed more negative emotions, including negative influence, intrusion, and criticism; correspondingly, their children showed higher levels of solute immunoglobulin A and greater social withdrawal [18]. In this study, immunoglobulin A levels were found to be associated with depression in Chinese older adults.

The mechanism through which immunoglobulins play a negative role in mental health is not clearly understood, but some studies have shown that immunoglobulins are related to neurotransmitter abnormalities [19]. Buranee found that immunoglobulins A and M could affect the regulation of central neural and glial activity through the tryptophan catabolite pathway by increasing the expression of picolinic acid and decreasing the concentration of quinolinic acid, which leads to physiological somatic symptoms [20, 21]. In addition, an increase in solute immunoglobulin A levels is not only a triggering factor of stress but also an aggravating factor for further stress. These mechanisms are related to the gut-brain axis and hypothalamic–pituitary–adrenal axis [22, 23]. Moreover, it has been reported that the paired immunoglobulin receptor B is related to inhibition of axon regeneration [24]. Secreted protein acidic and rich in cysteine-related protein containing immunoglobulin domains 1 could bind to pro-brain derived neurotrophic factor, negatively regulate its potential maturation, and affect brain nerve function, especially learning and memory processes [25].

This study has several limitations. First, as an observational and cross-sectional study, it cannot prove causality of the association between immunoglobulins and depression. Second, effective validation with further studies is necessary to determine the external validity or generalizability of our findings.

## Conclusions

The present study provides epidemiological evidence that immunoglobulin A levels have significant associations with depression in Chinese older adults. Further research should be conducted on the effects of regulating immunoglobulin A to improve depressive symptoms.

## Methods

### Participants

From June 2014 to December 2016, the CHCCS investigated older adults aged 80 years or older from 18 regions throughout Hainan, China. Based on a demographics list provided by the Department of Civil Affairs, the CHCCS enrolled a total of 1797 older adults [992 centenarians (55.20%) and 805 oldest-old adults (aged 80–99 years; 44.80%)]. All participants were community-dwelling older adults (Fig. 1a). The cohort profile and investigation method have been described previously [26]. The participants were assessed by neurologists who could communicate with them, and none of the participants had severe dementia or cognitive impairment, which made them unable to complete the GDS-15. After excluding 250 participants with missing GDS-15 (107 participants) or missing immunoglobulin levels (143 participants), the final analysis included data from 1547 participants [837 centenarians (54.10%) and 710 oldest-old adults (aged 80–99 years; 45.90%); Table 4]. The study protocol was approved by the Ethics Committee of Hainan Hospital of Chinese People's Liberation Army General Hospital (Sanya, China; Number: 301hn11201601). All participants gave their informed consent.

**Table 4:** Characteristics of all participants included and excluded in this study

Variables	Included (*n* = 1547)	Excluded (*n* = 250)	P
Age (year)^a^	94.75 ± 9.46	96.03 ± 9.33	0.068
Females (%)	1115 (72.07%)	184 (73.60%)	0.617
Ethnic Han (%)	1388 (89.72%)	225 (90.00%)	0.893
SBP (mmHg)^a^	150.91 ± 24.59	150.06 ± 25.45	0.637
DBP (mmHg)^a^	77.97 ± 13.40	77.61 ± 13.27	0.746
RBC (10^12^/L)^a^	4.20 ± 0.66	4.24 ± 0.65	0.504
Hemoglobin (g/L)^a^	119.03 ± 18.28	121.06 ± 20.42	0.482
MCHC (g/L)^a^	313.46 ± 13.13	314.78 ± 12.92	0.221
WBC (10^9^/L)^a^	6.21 ± 1.77	6.11 ± 1.98	0.098
Neutrophil^a^	0.56 ± 0.17	0.560.10	0.818
CRP (mg/L)^a^	0.57 ± 2.06	0.52 ± 0.92	0.925

*Notes*: ^a^ mean ± standard deviation

*Abbreviations*: *SBP* Systolic blood pressure, *DBP* Diastolic blood pressure, *RBC* Red blood cell, *MCHC* Mean corpuscular hemoglobin concentration, *WBC* White blood cell, *CRP* C-reactive protein

### Standard procedures

A baseline survey including epidemiological questionnaires, physical examination, and laboratory tests was conducted through a household survey of face-to-face interviews by the multidisciplinary research team consisting of geriatricians, neurologists, cardiologists, endocrinologists, nephrologists and nurses. All home interview surveyors were strictly trained, and physical examination were conducted following standard procedures [27, 28]. Age and sex were registered according to the second-generation identification card information of older adults. The operator measured the systolic and diastolic blood pressure of older adults who took a sitting position by electronic sphygmomanometers (Omron Hem-7200, Japan). Each parameter was measured twice and averaged, with at least a one-minute interval between the two measurements. The GDS-15 was used to measure depressive symptoms of the participants [29]. The scale had a maximal total score of 15 points and comprised 15 dichotomous items (possible range: 0–15) [29]. Depression was diagnosed by experienced neurologists based on the GDS-15 in combination with medical records and clinical symptoms by communicating with the participants and their family members. Scales > 6 were identified as potential depression, and higher scales indicated more severe symptoms of depression.

Samples of venous blood were obtained from all fasting participants by professional nurses and transported within 4 h in cold storage (4 °C) to the central laboratory. Red blood cell (RBC), hemoglobin, mean corpuscular hemoglobin concentration (MCHC), white blood cell (WBC), and neutrophil were detected by a blood autoanalyzer (SYSMEX XS-800I). Serum levels of immunoglobulin A, immunoglobulin G, immunoglobulin M, immunoglobulin E, complement C3, complement C4, and C-reactive protein (CRP) were determined by enzyme colorimetry (Roche Products Ltd., Basel, Switzerland) on a fully automatic biochemical autoanalyzer (COBAS c702; Roche Products Ltd.).

### Statistical analyses

Continuous variables are described as the mean ± standard deviation, which were compared using Student’s t tests. Categorical variables are described as percentages, which were compared using chi-square tests. Multivariate linear regression analyses were used to analyze the associations between immunoglobulins and depression. Multivariate logistic regression analyses were performed to determine independent correlates of depressive symptoms. All these were adjusted for in three models: Model 1: no adjustment; Model 2: adjusted for age, sex, and ethnicity; Model 3: age, sex, ethnicity, centenarians, systolic blood pressure (SBP), diastolic blood pressure (DBP), RBC, hemoglobin, MCHC, WBC, neutrophil, CRP, immunoglobulin A, immunoglobulin G, immunoglobulin M, immunoglobulin E, complement C3, and complement C4. Statistical analyses were performed with the SPSS 17.0 software package (Chicago, IL, USA). P values < 0.05 were considered statistically significant, and confidence intervals (CIs) were computed at the 95% level.

## Data Availability

The datasets used and analyzed during the present study are available from the corresponding author on reasonable request.

## Abbreviations

CHCCS: China Hainan Centenarian Cohort Study
CI: Confidence interval
CRP: C-reactive protein
DBP: Diastolic blood pressure
GDS-15: Geriatric Depression Scale
MCHC: Mean corpuscular hemoglobin concentration
OR: Odds ratio
RBC: Red blood cell
SBP: Systolic blood pressure
WBC: White blood cell
